# Supplementary material for: Pests, diseases and crop protection practices in the smallholder sweetpotato production system of the highlands of Papua New Guinea
Source: PeerJ. 2016 Dec 7;4:e2703. doi: 10.7717/peerj.2703 (PMC5147021; doi:10.7717/peerj.2703)
Supplement: Data S1 [file peerj-04-2703-s001.pdf]

Questinnaire	Enumerator	Surveyed village	region	province	where were you when you started talking to the farmer?
Prac000	Wills	KoreKore to	Asaro	EHP	Farmer's house
100	Willem	Kami 2	Lufa	EHP	Farmer's house
200	Deane Woruba	Kami2	Lufa	EHP	Village meeting
300	Kai LaLi	Kam2	Lufa	EHP	Farmer's house
400	Wills	Fogupavi	Lagaiee	EHP	Farmer's house
500	Wills	Lapane	Karuka	EHP	Farmer's house
600	Deane Woruba	Kuruka	Lufa	EHP	Farmer's house
700	Kai LaLi	Kuruka	Lufa	EHP	Farmer's house
800	Kai LaLi	KoreKore to	Asaro	EHP	Market
900	Deane Woruba	KoreKore to	Asaro	EHP	Village meeting
1000	Wills	KoreKore to	Asaro	EHP	neighboring village house
1100	Wills	KoreKore to	Asaro	EHP	meeting area of village
1200	Jeffery Yapo	KoreKore to	Asaro	EHP	Farmer's house

1300	Wills	Mu Yapokokil (Sina Sina)	Kamtai	Simbu	Distric office
1400	Deane Woruba	Kularemil , Sinasina	Kamtai	Simbu	Distric office
1500	Deane Woruba	Kularemil , Sinasina	Kamtai	Simbu	Distric office
1600	Jeffery Yapo	Munule, SinaSina	Kamtai	Simbu	Distric office
1700	Kai LaLi	Yapakure , Sinasina	Kamtai	Simbu	Roadside
1800	Wills	Jericho village	Kamtai	Simbu	Distric office
1900	Kai LaLi	Haina	Kamtai	Simbu	Roadside
2000	Jeffery Yapo	Haulabol, Gaima	Gumuni	Simbu	Farmer's house
2100	Deane Woruba	Omkaloai	Gumuni	Simbu	Village meeting
2200	Kai LaLi	Omkolai	Gumuni	Simbu	Village meeting
2300	Jeffery Yapo	Kel	Gumuni	Simbu	Farmer's garden
2400	Wills	Kawikul, Baul Dom	Gumuni	Simbu	Farmer's garden
2500	Kai LaLi	Mormul	Gumuni	Simbu	Farmer's garden
2600	Deane Woruba	Moro Maule	Gumuni	Simbu	Farmer's house
2700	survey lost before use				
2800	wills	Tambar	Hargen Central	Western Highland	Farmer's house

2900	Deane Woruba	Kombaip	Hargen Central	Western Highland	Farmer's house
3000	Kai LaLi	Tambor	Hargen Central	Western Highland	Farmer's house
3100	Wills	Lantiro	Hargen Central	Western Highland	Farmer's garden
3200	Jeffery Yapo	Tambra	Hargen Central	Western Highland	Famer's house
3300	Jeffery Yapo	Kelue	Hargen Central	Western Highland	Farmer's garden

Date of survey	Time start of survey	Date/tiem end of survey	Hours of survey	NEW<br>1:How long did it take to walk to the garden (min)	GARDEN<br>2. What was the terrain like to walk to this garden?	3: Latitude	3:Longtitude
1/27/2014 3:30:00pm	15:00:00	17:15:00	02:15:00	15	sloping, rugged	6 00 39.1 S	145 18 06.1 E
1/28/2014 8:50am	8:50 AM	11:00 AM	02:10:00	15-20	sloping	6 17 7 S	145 25 3 E
1/28/2014 9:45:00 AM	9:45:00 AM	12:20:00 PM	02:35:00				
1/28/2014 9:33:00am	9:33:00 AM	12:40:00 PM	03:07:00	3	sloping		
1/28/2014 2:03:00	2:03:00 PM	2:42:00 PM	00:39:00				
1/29/2014 9:17:00 am	9:17:00 AM	10:37 AM	01:20:00	20-30	rugged	6 20 24.4 s	145 21 57.2 E
1/29/2014 9:30:00 AM	9:30:00 AM	1:30:00 PM	4:00:00	20	Steep	6° 2' 45" S	145° 1' 07" E
1/29/2014 9:15:00 am	9:15:00 AM	11:56:00 AM	2:41:00	60	sloping		
1/30/2014 9:44:00 am	9:40:00 AM	11:37:00 AM	1:57:00	15	Flat		
1/30/2014 9:55:00 am	9:55:00 AM			40	sloping		
1/30/2014 9:44:00 am	9:44:00 AM	11:42:00	1:58:00	40	Steep		
1/30/2014 11:45 am	11:45:00 AM	12:46:00 PM	1:01:00	20	Flat	6 01 15.7 s	145 18 29.7 E
1/30/2014 9:44:00 am	9:44:00 AM	11:42:00 AM	1:58:00	15-18	sloping		

1/31/2014 11:07:00 am	11:07:00 AM	12:04:00 PM	0:57:00	2	sloping	6 5 23.o S	145 06 06.9 E
1/31/2014 11:20:00 am	11:20:00	12:00:00 PM	0:40:00	10	sloping	6 5 16.6 S	145 2 3.3 E
1/31/2014 12:00:00	12:00:00	12:45:00 PM	0:45:00	10	sloping	6 5 32.2 S	145 1 57.6 E
1/31/2014 11:09:00 am	11:09:00 AM	12:30:00 PM	1:21:00				
1/31/2014 11:05:00 am			0:00:00	2	sloping		
1/31/2014 12:07:00 pn	12:07:00 PM	1:50:00 PM	1:43:00	5	sloping	6 05 38 S	145 01 14 E
1/31/2014 12:09:00 pm	12:09:00 PM	12:49:00 PM	0:40:00	3	sloping		
3/2/2014 11:49	11:49:00 AM	12:52:00 PM	1:03:00	35	sloping		
3/2/2014	1:00:00 PM	1:45:00 PM	0:45:00	20		6 11 2.9 S	144 56 47.6 E
3/2/2014 12:53	12:53:00 PM	2:32:00 PM	1:39:00	3	sloping		
4/2/2014 10:14	10:14:00 AM	11:30:00 AM	1:16:00	14	sloping		
4/2/2014 10:51	10:51:00 AM	12:50:00 PM	1:59:00	5	steep	6 11 4 S	144 56 52 E
4/2/2014 11:09	11:09:00 AM	12:41:00 PM	1:32:00	3	sloping		
4/2/2014 11:20	11:20:00 AM	12:00:00 PM	0:40:00	20	sloping	6 7 13.1 S	144 55 31.9 E
6/2/2014 10:05	10:05 AM	11:14 AM	1:09:00	2	Flat	5 46 36 S	144 14 58 E

6/2/2014 10:15	10:15 AM	11:15 AM	1:00:00	20	sloping		
6/2/2014 9:40	9:40 AM	12:10 PM	2:30:00	5	Flat		
7/2/2014 9:16	9:16 AM	10:00 AM	0:44:00	0	flat	5 49 32 S	144 18 57 E
6/2/2014	10:09:00 AM	10:56:00 AM	0:47:00	10	Flat		
7/2/2014 11:25	11:25 AM	12:13 PM	0:48:00				

3: Altitude	6.How many years was this garden under fallow last time?	Years for mean	13. How long since this land came out of the fallow? (years)	14. How many plantings of sweet potato have there been in this new garden?	15. How long ago were the sweet potatoes in this garden planted? (month)	17. What do you think the sweet potato yield will be in this garden?
1628	0.25	0.25	3	1	3	low
1610	50+	50	0.08	1	1	high
	4	4	5 years	2	3	medium
1950	2	2	5 mo	1	5	high
2042	9	9	9		3 month ago	high
	5-6	5.50	5-6 years	1	3 month ago	high
	1-2	1.5	2 years	2	3 month ago	medium
	1	1	1 year	1	3 month ago	high
	30+	30	12	1	5+	high
1558	3	3	4mo	1	4 mo	high
	4 mo	0.33	3 mo	1	3 month ago	high

1955	10+	10	7 mo		1	5+	high
1962	11	11	2 mo		0		high
1945	20+	20			1	5+	high
	5 mo	0.42	5mo		50	5mo	high
	2	2	6mo		1	4mo	medium
1934	0.5	0.5	1 yr		2	3mo	low
	1	1	1.5 yr		1	more than 5 mo	medium
	10	10	6mo		1	2mo ago	high
1592	0	0		3	2	5mo	high
	0.5	0.5			3	3mo	low
	20+	20		6	1	more than 5 mo	medium
1554	0.25	0.25	2-3 month		1	3 month ago	medium
	20	20	16-18 mo		1	2mo ago	high
1726			1 year		1	3 month ago	high
1749	2	2		3	1	3 month ago	high

		6	6	1	1	3 month ago	high
		1	1	3	2	3 month ago	medium
1646	6-8		7	2	1	2 mo	high
		1	1	4	1	4 mo	high
			7.560345				

28. Which part of sweet potato plants get attacked by pests?	28: local name and type of pest they have noticed, the incidence of attack and severity	29. What time of the year or cropping cycle are the pests most active? Make separate note for each type of pest named above.
stem, leaves, tubers	SP weevil: attach when potato is ready, cricket: as soon as they plant	Pest are always there depends on age of crop that it then attacks
tuber	SP weevil: Sep-Dec, Criket; Dec-Aug	SP weevil: Sep-Dec, Criket : Dec-Aut
tuber	weevil during dry season	during maturity period of SP and dry season
tuber	weevil during dry season	weevil: dry season, may-june
no		does not have pest problem
stem, leaves, tubers	cricket, cut worm, insect attack new gardens	June-Sept
stem, leaves, roots, tubers	snails, cricket, weevils	may-august
tuber	cricket: slight damage, weevil: dry season, major damage	criket: wet season, weevil: dry season
stem leaves, tubers	weevil, dry season	criket: wet season, weevil: dry season
stem, leaves, tubers	SP weevil: during dry season, major yield loss, ants: attacks newly planted vines	all year around
stem, leaves, roots, tubers	weevil (senegaha): all year around, major yield loss,; rat: reduce crop yield	weevil: active when crop is 4 month old

stem, leaves, tubers	weevil, dry season, major yield loss	weevil, druing dry season (june-Aug)
root, tubers	weevil	weevils in dry season
stem, leaves, roots, tubers	catapillar type, found in fresh; grasshopper, eat leaves	catapillar: 5-6 mo when SP become mature, grasshopper: all year around
stem, leaves, tubers	when crop is young and close to harvest	all year around
tubers	weevil, dry season, small yield loss	weevil, during March to July
stem, leaves, roots, tubers	weevils, scab, gall mite	scab, gall mite at yielding stage, weevil during dry season
stem, leaves, tubers	weevil: spoil flesh, all year aroudn	weevil: at maturity stage, 2-6 months
stem, leaves, roots, tubers	weevil: eat tubers, criket, eat leaves; small black snake, cut roots	weevil: dry season; criket: all year around; small black snake, during new planting
stem, leaves, roots, tubers	weevil, scab	after 2-3 month of planting
stem, leaves, tubers	grasshopper, damdom (?), weebil	all pests are active as soon as crop put leaves (>2mo)
stem, leaves, tubers	weevil, criket, yellow catarpillar, all year around	all pests occur all year around
stem, leaves	unknown insects attacking leaves	when SP are 2-2.5 mo old
stem, leaves, tubers	weevil	weevil attack during dry season
tubers	weevil, dry season, small yield decline;	weevil, all year around

tubers	weevils	all year around
stem, leaves, tubers	leaves damaged by scabs, oribius, hopping insects after 2-3 month old	no specific time or month
stem, leaves, tubers	caterpillars: all year around though damage is insignificant, weevil: during dry season, major yield loss	unpredictable
stem, leaves, tubers	pests are present, reduce yield, when SP is 3-5 month old	when crop is 3-5 monts old

30. What action do you take to control or prevent these types of pests? Make separate note for each type of pest named above.	31. Which part of sweet potato plants get attacked by plant diseases?	31:description using local names the types of diseases thay have noticed, the incidence of attack and severity
none	stem and leaves	Gall mite & Scab: occurs all year around but common during wet season, no idea how it affects crop yield
no action taken. Ants to control the weevil.	roots	tubers become soft with no reasons
no : don't know how and NARI can help me	no	no disease but during dry season all SP normally die
no control or prevention	stem and leaves	occur in all year around, not severe, low impact on yield
nothing	no	
no	roots, tubers	leaves turned yellow and tubers rot
purchase chemicals from store and spray (KARATE = name)	no	
do nothing	stem and leaves	gall on leaf
no control method	stem and leaves	
no control or prevention	stem and leaves	scab and gall mite, all year around
remove infested vine; for rat, cover the exposed vine	leaves and tuber	

no control method	stem and leaves	scab and gall mite, all year around
do nothing	stem and leaves	galls mite
harvest as soon as they find	stem and leaves	no name: black dots on the skin, swollen skin
no control method	stem, leaves, roots, tuber	once tuber is formed, vines rot and affect yield
breaking up mounds to expose tubers to the heat	tubers	Gall mite & Scab: occurs all year around but common during wet season, no idea how it affects crop yield
no control		
no control	leaves and tuber	scab and gall mite
do nothing	stem, leaves	gall mite, catapillars
do nothing	stem, leaves, tuber	leaf rolls
biological control - use chicken	stem, leaves, roots,	gall mite, scab
no control	stem, leaves,	rust, scab, gall mite
no action taken.<br>use piper leaves, Kom grass leaves, and fish lass leaves (tephroshia) on surface of mound to control SP weevil	stem, leaves,	galls mite
patting up of soil under the SP vine where the tubers are	tubers	gall mite

cover tubers well	stem leaves	leaves crumbled up occasionally when SP are at the age of 203 months and reduce tubers numbers and weight drops
very hard to control	tubers	
no action taken	stem leaves	gall mite: all year around, planting materials are affected
no action taken	stem, leaves, tubers	unknown disease, reduce yield

32. What time of the year or cropping cycle are the diseases most active? Make separate note for each type of disease named above.	33. What action do you take to control or prevent these types of diseases? Make separate note for each type of disease named above.	35. Is the planting material you use certified, disease tested or special in any way?
occurs throughout the year	choosing planting materials not affected by the disease	no
unseasonal	no control /prevention method	no
		no, just from old garden
all year around	no control/prevention	no, just from old garden
		no
Oct-Jan	leave it fallow and go to new garden	nil
		no, just from old garden
diseases most active during rainny season	do nothing	no
all year around	no control /prevention meth od	no it isn't
all year around	no control /prevention meth od	no
gall mite, scab at 2-5 month time	no control /prevention meth od	no

scab and gall mite occur 3-4 month after planting	no control /prevention meth od	no
all year around	break off galled stem and leaves	no
maturity of tuber	no control/prevention	no
first tuber formation to middle age of harvest	no control/prevention	no
gall mite, all year around	no control /prevention meth od	no
		no
during the harvesting time	no control	no
gall mite, cattapillar, all year around	do nothing	no
when SP are in the mid-maturity age	do nothing	nil
during the maturity stage	do nothing	no
all year around	no	no
during the rainy season	manual removal of gall-affected leaves/stem	no
all year around	no control	no

leaves crumbled up - always present, all year around	do nothing	no
all year around	nil	nil
all year around	no control	no
3-5 month old	no control	no

38. List your top three concerns about sweet potato pests or diseases; what problems do most want fixed, new research to help grown healthier crops?	46: Check the tubers for insect holes (weevils or other grubs). location 1	46 location 2	47: Check the tubers and rate how many insect holes there are, location 1
weevil			
weevil, unexplained softing tubers			
weevil, criket, scales			
weevil			
none			
weevil, nematode, insect			
improving soil fertility, control insects	none	none	none
weevil, criket			
weevil, criket		3-8 mm	
weevil			
weevil, scab/gall mite, rat	none	none	few just detectable

weevil, scab, mite	less than 3mm	none	obvious
new variety to resist pest; new research for method to control pest; need awareness weevil in dry season, low yield reduce tuber, repeating same land	none	none	none
weevil, gall mite			
weevil, yield decline, soil improvement	between 3-8 mm	less than 3 mm	many holes in tubers
scab, weevil, gall mite			
weevil	none		
scab, weevil, soil fertility	none	less than 3 mm	few just detectable
new technologies, new varieties	less than 3mm	between 3-8 mm	many holes in tubers
weevil, rust, grass hoppers			
scab, slow growth, insects			
weevil, gall mite	less than 3mm		few just detectable
weevil			

weevil	
scab, weevil, soil fertility	
weevil, gall mite	
soil fertility, new variety of SP, sustain crop productivity	

47: location 2	48: Split open the base of the stem of the sweet potato plant and check for the presence of weevils or borers in the stem, location 1	48: location 2	49: What name do you call this variety of sweet potato? location 1
			I don’t care
			Eana
			Kenba
			Marasota
none	none	none	carrot
			I don't care
few just detectable		none	
			I don't care
few just detectable	none	none	I don't care

none	none	none	susan's black eye
			wagi besta
			carrot kaukau
none	none	none	wagi besta
			wagi besta
many holes in tubers	larvae present	larvae presnt	susan's black eye
	none		I don't care
few just detectable	none	none	Sugar
many holes in tubers	tunnels visible	tunnels visible	wagi besta
			Goroka guy
			I don't care
	tunnels visible		I don't care
			Korowes

Rachel's found

Baya Makep

cheese pop

49: location 2	50: How old (months) is the plant that was dug up? location 1	50: location 2	51: Have tubers already been harvested from this plant? location 1	51: location 2	52: Soil sampling (0-10 cm)	55a: Healthy plants with large dark-grean leaves and dense canopy	55b: all leaves smallish and light or olive green	55c: Oldest leaves yellowing or drying up
I don't care	2	2	no	no		y		
Marasoa	1	1	no	no				y
Wagi besta								
Wagi besta	1mo	1mo	no	no				
						y		
gimane	2	2	no	no		y		
I don't care	3 mo	3 mo	no	no			y	y
carot kaukau		7		2 times		y		
I don't care	4	4	1	1				y
I don't care	3	3	no	no				

Okangai	1 mo		none	once		y	
Wagi besta	3	3			y		
Susan's black eye	5	5	no	no	y		
Susan's black eye	4	4	n	no	y	y	
gaigol	4	4	no	no			
Susan's black eye	6	6	1	1		y	y
					y		
I don’t care	5	5	0	3			y
Goroka Guy	3	3	no	no	y		y
Wagi besta	6	6	4	4	y		
Wagi besta	2	2	no	no			
Wagi besta	2	1.5	no	no			y
I don’t care	7	7	3	3	y		
Korowes	3	3	no	no	y		

Rachel's found	7	7	4	4	y
3 month SP	2	2	0	0	
sugar	3	3	no	no	y
					y

55d: Older leavres red or orange before yellowing or drying up	55e: light green mottling	55f: deformities of young leaves	55g: leaves dropping off in the middle of the viene	55h: other	OLD<br>56. Estimate the area of one mound or bed.	GARDEN<br>57. How many plants are in one mound or bed?	101. How long did it take to walk to the garden? (min)
					0.7x1	2	0
					0.4-0.7 x 0.4	2	15-20
							5
					0.8 x 0.8	10	5
							0
				leaves with holes many grass hopper, no significant damage	0.8 x 0.8	1-2	20-30
					0.5 x 0.5	2-3	60
					0.8 x 0.8	3	
y	y	y			0.8 x 0.8	3	
		y			1 x 0.6	3	2
					0.6 x 0.6	2	10
					0.5 x 0.5	2-3	

			y		0.8 x 0.8		3	2
								10
					0.9 x 0.9		3	
y		y			0.75 x 0.75		3	20
y					1 x 1		3	2
y		y			0.75 x 0.75		3	5
y			y		1 x 1	2-3		2
		y		gall mite and leaf scab	0.6 x 0.6	2-3		40
y	y	y			1 x 1		3	30
					0.9 x 0.9		3	3
		y			0.6 x 0.6		2	24
		y			0.8 x 0.8		2	5
			y		0.8 x 0.8		2	5
					1 x 1		3	20
		y			2 x 1.5		2	2

			0.3 x 0.3		3	20
			0.9 x 1.2		14	5
			0.3 x 3		2	0
		Gall mite attach	4 x 4	15-20		15
						3

102. What was the terrain like to walk to this garden?	103 Latitude	103 Longitude	103:altitude	108. How many years was the garden under fallow last time?	years	117. what do you think the sweetpotato yield will be in this garden?	128. Which part of sweet potato plants get attacked by pests?
sloping	6 00 39.1	145 18 06.1		too long ago		low	stem, leaves, tubers
sloping	6 17 7 S	145 25 3 E	1610	1	1	low	tuber
sloping	6° 17.0925'S	145° 25.3601'E		never		medium	The tubers
sloping				6	6	don't know	stem, leaves, roots, tubers
flat				40+	40	low	tuber
rugged	6 20 24.4	145 21 57.2	1910	40+	40	medium	tubers
sloping				3-4	3.5	low	stem, leaves, roots, tubers
flat						low	stem leaves, tubers
flat	6 01 15.7	145 18 29.7	1558	3	3	low	stem leaves
						low	stem, leaves, roots, t ubers

sloping	6 5 23	145 6 6.9	1955	10+	10	low	stem, leaves, tubers
				never		medium	root
sloping				3	3	medium	tubers
sloping	6 5 45 s	145 1 12 e	1903	0.5	0.5	low	stem, leaves, roots, tubers
sloping				0		medium	stem, leaves, roots, tubers
sloping	6 5 38 S	14 1 14 E	1934	2	2	low	tubers
flat				3	3		stem, leaves, roots, tubers
steep				10	10	low	
sloping				1	1	low	stem, leaves, roots, t ubers
sloping						low	roots, tubers
sloping				0.5	0.5	low	stem, leaves, tubers
sloping	6 11 4 S	144 56 52 E	1554			low	stem, leaves, tubers
sloping				20+	20	low	stem, leaves, roots, tubers
sloping	6 7 13.1 S	144 55 31.9 E	1726	4	4	high	stem, leaves, tubers
flat	5 46 36 S	144 14 58 E	1749	?		high	tubers

sloping				6	6	low	tubers
sloping				6 mo	0-Jan		stem, leaves
flat	5 49 32 S	144 18 57 E	1646	6 mo	0.5	medium	stem, leaves, tubers
flat				6-7 mo	0.59	low	stem, leaves, tubers
flat				3 mo	0.25	high	stem, leaves, tubers
					7.39714		

128: describe using local names the types of pests they have noticed, the incidence of attack, and severity	129. What time of the year or cropping cycle are the pests most active?	130. What action do you take to control or prevent these types of pests?
SP weevil: attach when potato is ready, cricket: as soon as they plant	Pest are always there depends on age of crop that it then attacks	no action
SP weevil: Sep-Dec, Criket; Dec-Aug	SP weevil: Sep-Dec, Criket : Dec-Aut	Ants to control the weevil.
sweetpotato weevil during dry season (Apr - Sep)	Weevil: during wet season	None
leaf scabs, weevil	leaf scab: 3-4 mo old, weevil: when tubers are formed and during dry season	None
weevil: so serious, always exist, high yield loss, Cricket: less serious and small yield loss	weevil: all year around, Criket: all year around	no control/prevention method
weevil: high yield loss, especially in dry season	weeble: dry season, May-June	no control/prevention method
weebel, scabs, criket	weevil: dry season, scaves	no control/prevention method
weevil during dry periods from june to August, crikets in all year around	weevil: dry season, criket: all year around	no control/prevention method
weevil: dry season, high yield loss	weevil: dry season, high yield loss	no action
weevil (senegaha): all year around, major yield loss,; rat: reduce crop yield	weevil: active when crop is 4 month old	remove infested vine; for rat, cover the exposed vine

weevil, dry season, major yield loss	pest is active during dry season	no control/prevention method
	during dry season, but now even in wet season, weevil still attack	do nothing
weevils, yield loss	weevil, during dry season	do nothing
catapillar type, found in fresh; grasshopper, eat leaves	catapillar: 5-6 mo when SP become mature, grasshopper: all year around	harvest as soon as they find
affectd from scab	August-october	nothing
weevil, during dry season, small yield loss	weevil, druing dry season, from March to July	weevil, expose SP to heat
weevils, scab, gall mite	scab, gall mite at yielding stage, weevil during dry season	no control
weevil in dry period, many unknown species	2-6 mo when start producing tubers	no control
weevil, major yield loss	weevil: dry season	put tefroshia and piper leaves on mound
scab, weevil	when SP is 2 mo old	do nothing
grasshopper: eat leaves and tubers; giant snail (damdam): eat leaves; unknown pest	all cause major damage in 3-5 month old plants	no action
weevil, criket, yellow catarpillar, all year around	all pests occur all year around	no control/prevention method
scab, weevil	all year around but close to harvest and 2-3 month are bad	cro rotation
weevil, gall mites	both all year around, but weevil is worse in dry season	use fish-kill (tefroshia) in natural fertiliser mix, or pipe leaves
weevil, dry season, small yield decline;	weevil, all year around	no control method

weevils, all year around	all year around	make sure tubers are properly covered
scab	scab attacks when SP is 2-3 month old	no control
caterpillars: all year around though damage is insignificant, weevil: during dry season, major yield loss	unpredictable	no control
pests are present but don't know what they are	when crops are 2-5 month old	no control
Oketepna: during dry season (probably weevil), major yield loss	dry season	no control

131. Which part of sweet potato plants get attacked by plant diseases?	131: describe using local names the types of diseases they have noticed, the incidence of attack, and severity	132. What time of the year or cropping cycle are the diseases most active?
stem and leaves	same disease in new gardens affect the crop	occurs throughout the year
roots	tubers become soft with no reasons	unseasonal
stem, leaves	Galls: appeard throughout cropping cycle in some varieties. Not observed lately. Farmer believes this is due to non-selection of sustainable variety	Galls: mostly during wet season, Scab: mostly during wet season
tubers	SP weevils are infested in the tubers. smell com out from the tubers when harvested	during dry season
tubers	unexplained softing of tubers	no seasonal difference
stem and leaves	no serious disease	occasionally
stem and leaves, roots, tuber	yield drop,	dry seasons and maturity of the crops
stem and leaves	scab and gall mite	all year around
stem leaves	scab an gall mite, all yaear around, insignificant impact on yield	all year around
leaves and tuber		gall mite, scab at 2-5 month time

stem leaves	scab an gall mite, all yaear around, insignificant impact on yield	scab and gall mite occur during the growth of the crop
no		dry season -weevil damage prominent
stem and leaves	Galls:	all year around
stem and leaves	scab and gall mite	gall mite
stem and leaves	gall mite, all year around, insiginificant yield loss	gall mite, all year around
stem, leaves, tubers	gall mite, scab	2-4 mo old of vines
stem, leaves	gall mite, scab; both become more frequent	during wet season
stem, leaves, roots, tubers	gallmite, scab; 2-5 month old plants	when plants are 2-5 month age
stem, leaves,	rust, scab, gall mite	all year around
roots	rotton tuber	when SP is mature and wet season
stem and leaves	galls, scab	they become more frequently, especially in wet season
tubers	gall mite	all year around

stem, leaves	scab in leaves	all year around
stem leaves	gall mite: all year around, planting materials are affected	all year around
stem, leaves, tubers	gall mite, scab, all year around, major yield loss	all year around
stem, leaves, tubers	gall mite: worse when crop get mature, yield loss	when crop is 2-5 month old

133. What action do you take to control or prevent these types of diseases?	134. From where do you obtain your planting material of sweetpotato?	135. Is the planting material you use certified disease tested or special in any way?	136. What is the typical length of time between sweet potato crops
slection of clean material	own gaden	no	1 mo
no control /prevention method	from own garden	no	1 week
choose sweetpotato variety resistant /tolerant to disease	from own gardens	no	6 mo plant once a year, plant other crop, then plant sweetpotato again
do nothing	from the bush gardens	no	3 mo
no control /prevention method	from own gardens	no	varies betwen 1-3 mo
no control /prevention method	from own garden	no	1 mo
nil	from the old gardens	no	4-6 years
no control /prevention method	from own garden	no	1 mo
no control /prevention method	from own garden	no	1 mo
no control /prevention method	from old garden	no	4-5 weeks

no control /prevention method	from owan garden	no	1-2 mo
no control /prevention method	from own gardens	no	2-3 mo
no control /prevention method	from own garden	no	2 mo
no control /prevention method	from old garden	no	6-7 yrs interval=unknown
	from old garden	no	6mo
no control /prevention method	from own gardens	no	1 mo
	from own garden	no	8-9 mo
no control /prevention method	from old garden	no	2 mo
do nothing	from own gardens	no	2mo
do nothing	from old garden	no	10-20 years
no control /prevention method	from own gardens	no	1 mo
no control /prevention method	from own gardens	no	3 mo
no control /prevention method	from own gardens	no	2mo
no control /prevention method	from own gardens	no	1 mo

do nothing	from own gardens	no		0
	from own gardens	no		4
no control /prevention method	from family members	no	1 mo	
no control /prevention method	old garden	no	10-20 years	
no control /prevention method	old garden	no	20-30 years	

137. Do you prepare the land for sweet potato planting in any special way to help prevent pest and disease problems	138. List your top three concerns about sweet potato pests or diseases; what problems do most want fixed, new research to help grown healthier crops?	146: Are thre insect holes in tubers? locaiton 1	146: location 2	147:Check the tubers and rate how many insect holes there are, location 1
no	weevil			
no	weevil, unexpecting softening			
no	Weevil, gall, scab	<3mm	none	obvious
no	scab, weevil, loss of yield	<3mm	<3mm	Many holes in tubers
no	weevil control, criket control			
no	weevil	none	none	none
no	weevil		<3 mm	
no	weevil	none	none	none
no	weevil			
no	weevil, scab/gall mite, rat			

no	weevil, scab, gall mite			
no	weevil			
no	weevil, galls	none	less than 3 mm	none
no	pest control, new variety for pest registant and quick growth			
no	weevil, scab, gall mite			
no	weevil, gall mite			
no	soil fertility, weevil	less than 3mm		few just detectable
no	scab, gall mite, weevil	none	none	none
no	weevil			
		none		few just detectable
no	new technology, new variety		less than 3 mm	
no	weevil, fungal disease			
no	weevil, leaf scab, tubor rot	less than 3mm	larger	none
no	weevil	less than 3mm	less than 3 mm	few just detectable
yes, eartting up at the SP base during tuberization of the crop,	weevil			

no	weevil			
no	scab, weevil, soil fertility			
no	weevil, gall mite	less than 3mm	less than 3mm	few just detectable
no	new technique to resist pests and diseases, new varieties		less than 3mm	
no	gall mite, weevil, new variety to regist pest and diseases			

147: location 2	148: Split open the base of the stem of the sweet potato plant and check for the presence of weevils or borers in the stem. location 1	148: location 2	155a: Healthy plants with large dark-grean leaves and dense canopy	155b: all leaves smallish and light or olive green	155c: Oldest leaves yellowing or drying up	155d: Older leavres red or orange before yellowing or drying up
			y			
one	Tunnels visible	none		y	y	
many holes in tubers	larvae present	larvae presnt				
			y			
none	non	none	y			
just detectable		none	y	y		
none	non	none		y	y	y

				y	y	
obvious, easily visible	non	larvae present		y	y	y
						y
			y		y	y
	larvae present					
none	non	tunnels visible				
f	larvae present	larvae present				
obvious, easily visible		tunnels visible			y	
many holes in tubers	larvae present	larvae present				y
few just detectable	none	none		y	y	y

				y	
few just detectable	none	none		y	
obvious, easily visible		larvae present		y	y
			y		

155e: light green mottling	155f: deformities of young leaves	155g: leaves dropping off in the middle of the viene	155h: other
	y		low scab infestation, holes in leaves
	y		visible weevil & scab damages
			small number of leaves have insect damage
	y		
	y		call mite and scab

	y		ga;;s amd scab
y			
	y		less dense canopy over the time
			young and old leaves have holes by grasshopper
	y		leaves damaged by grasshopper
	y		
		y	
y	y		

	y
	y
